# Development and Evaluation: A Behavioral Activation Mobile Application for Self-Management of Stress for College Students

**DOI:** 10.3390/healthcare10101880

**Published:** 2022-09-27

**Authors:** Hyunjoo Na, Minjeong Jo, Chaerin Lee, Doyoon Kim

**Affiliations:** College of Nursing, The Catholic University of Korea, Seoul 06591, Korea

**Keywords:** mobile health application, participatory research, behavioral activation, psychological intervention, stress management

## Abstract

College students are at a high risk of mental health problems due to continuous exposure to considerable stress as they transition into adulthood. It is necessary to reflect on young people’s needs and provide brief, personalized support interventions via mobile applications. This study aimed to (1) describe the co-design development process of a behavioral activation (BA) mobile health application called MEndorphins to help youth manage stress; and (2) evaluate the ease of use and quality of the application and its effects on psychological distress. College students aged 18–25 in South Korea participated as co-designers throughout the MEndorphins development process, which involved prototyping workshops. Thirty-five students also evaluated the application’s ease of use and quality, as well as its effects on psychological distress, using a self-reported online questionnaire. In the pilot evaluation, ease of use scored 74.21 out of 100 and quality 3.72 out of 5. There were statistically significant decreases in depression, anxiety, and stress after using MEndorphins (*p* ≤ 0.001 for depression and anxiety, *p* = 0.001 for stress) for 7 days. In this developed BA based mobile application, participants could monitor their mood, plan stress self-management strategies, and gain motivation by sharing experiences.

## 1. Introduction

One in four South Koreans experiences a mental health issue at least once in life, and those aged 20 to 29 years are at an especially high risk of mood and substance abuse disorders [1]. Most Korean high school students choose to enter undergraduate programs in college, except in special cases such as early employment. Thus, college students make up a large proportion of the population in their 20s. College students are in the transitional period to adulthood. During this period, they are asked to accomplish a variety of tasks such as establishing an identity, becoming independent from their parents, maintaining a successful college life, and choosing a career [2]. Many young Koreans in their 20s face fierce competition during their high school years as they prepare for entrance exams to enter college, and upon entering college, they immediately start focusing on preparing for employment, such as taking up language studies or internships. These circumstances often cause significant stress in Korean college students.

Stress has been shown to negatively impact college students’ academic performance, as well as their sleep quality, anxiety, depression, and physical health [3,4]. Continuous exposure to high levels of stress during academic life eventually leads to the deterioration of college students’ quality of life. Therefore, it is necessary to properly manage stress to alleviate its negative consequences. Various treatments have been developed for college students to manage stress [4], and these services are usually delivered in groups through face-to-face counseling or education [5]. However, since the outbreak of the COVID-19 pandemic (the coronavirus disease 2019) in 2019, college students have been facing difficulties participating not only in classes but also in face-to-face counseling for stress management [6]. In such circumstances, an alternative means of support is necessary to help students gain access to psychological distress support.

Advances in mobile technology (e.g., smartphones and handheld computers) have provided people with easier access to mental health services. Today’s 20s are a generation that is familiar with digital culture. They were born under the ubiquitous influence of the Internet and mobile devices [7,8]. College students may want to manage their stress in an easier and simpler way than face-to-face meetings, which require rather complicated procedures such as appointments or transfers to clinics. Considering the attributes of the younger generation, providing interventions for stress management via mobile phones, often referred to as mobile health (mHealth) interventions, may be more effective.

Among mental health management approaches, behavioral activation (BA) has been used widely to promote behavioral change [9]. BA interventions focus on alleviating an individual’s exposure to sources of negative reinforcement such as stress, while increasing exposure to experiences of positive reinforcement (i.e., activities that bring positive emotions and achievement) and preventing future relapses of avoidant-type behaviors [10]. This suggests that BA can be a therapeutic approach that can motivate and strengthen college students’ independent coping skills and can reduce negative emotional responses such as stress and psychological problems consequently [11]. BA techniques can help young people to motivate positive behaviors and experience pleasure and achievement through the planned activities. The BA approach has also been used to develop a mobile application (hereafter, “app”) content for stress management and useful tracking behaviors, and to recommend appropriate coping strategies [11]. Considering that college students are at ease with mobile phone use, an app informed by the BA approach is expected to help them deal with stress and the consequent psychological distress. Therefore, in this study, a mobile app was developed using a co-design process so as to incorporate the perspectives of college students in their 20s, and evaluated the usability and quality of the developed app, as well as its effects on psychological outcomes.

## 2. Methods

This study consists of two parts. In Section 2.1, the process of mobile app development with co-design was described. In Section 2.2, the details of our evaluation of the usability and quality of the developed app, and its effects on psychological outcomes were summarized. Ethical approval for this study was obtained from the institutional review board of a university. The study protocol was registered with the International Standard Randomized Controlled Trial Number registry (ISRCTN12017266).

### 2.1. Development of App

The mHealth app for self-management of stress using a participatory co-design was developed based on the guidelines of Young and Well [12]. The guidelines comprise the following steps: (1) content development, (2) content validity evaluation, and (3) co-design workshops for prototyping.

#### 2.1.1. Content Development

The primary investigators (PIs) are composed of a student committee for content development. Five nursing college students (CL, DK, JK1, JK2, and JL) were recruited as committee members for app development to reflect their real-life stressors and identify appropriate stress management strategies among nursing students. The committee’s roles included creating and evaluating the app content, and organizing workshops under the supervision of the PIs. The PIs and the committee named the mHealth app to be developed in this study MEndorphins. MEndorphins are a combination of mental health and endorphins. The name was considered suggestive of the main function of the proposed app, which was stress management. Studies published between 2010 and 2019 on BA-based mobile apps to reduce stress were searched and reviewed. The reviewed components were assessment tools, contents and methods of intervention, and main results. In addition, Korean and English mobile apps that were released between 2016 and 2019 on Android and IOS platforms were reviewed. The reviewed items included the app names, target users, features, and main functions.

Based on the review, the PIs and the committee decided to apply the BA techniques to four main content components: monitoring, planning, feedback, and sharing.

Monitoring: The purpose of monitoring was to clearly and immediately recognize the students’ current mood or psychological status through the mobile screen. The PI and committee developed eight stress categories that were considered to occur mainly in participants in their 20s. The categories included worries about academics, finance, the future, life values, relationships with friends, family, lovers, and professors, which are based on the stressors suggested by the Revised Life Stress Scale for College Students (RLSS-CS) [13,14].

Planning: The purpose of planning was to help students plan their stress management by suggesting easy strategies that can reduce stress and increase positive behaviors. Stress management strategies that are expected to be primarily used by college students were developed. They consisted of the following five categories: relaxation therapy, artistic activities, physical activities, finding supportive relationships, and problem-solving. These strategies were developed based on the list of pleasant and easy activities suggested by Davis et al. [15] in the Tactics for Coping Stress Inventory. Approximately 9 to 10 example strategies were registered for each stress management category. For instance, in the “artistic activities” category, strategies such as “visiting museums and recitals,” “appreciating and recording beautiful moments of the day,” and “laughing while watching TV or YouTube” were suggested. Students could also implement stress management strategies that they personally desire by entering specific strategies of their own.

Feedback: The purpose of feedback was to enable students to self-evaluate the behaviors they actually implemented, that is, the stress management techniques they used. The feedback would indicate changes in the level of stress after performing the planned activities and completing strategic activities each day. Additionally, feedback on the stress-coping strategies that were implemented by the students most effectively was also considered. With these components, students could obtain feedback on stress management in various ways, enabling them to identify the strategies that work best for them. A push-alarm function was also planned to allow participants to set an alarm at a specific time to motivate them to perform the planned positive behavior.

Sharing: The purpose of sharing was to help students share their experiences and gain information about new, effective stress management strategies from others of their age. To this end, the committee proposed to develop an online community where students who use the developed app can share app experiences and strategies for stress management.

#### 2.1.2. Content Validity Evaluation

After the app content components were determined, the content validity index (CVI) was evaluated by a panel of four mental health experts: a psychiatric physician, two professors in mental health nursing, and a psychologist. Each expert rated the relevance of the individual items on a four-point scale (1 = not relevant, 2 = somewhat relevant, 3 = quite relevant, and 4 = highly relevant) [16]. The CVI for the developed content ranged from 0.75 to 1.0.

#### 2.1.3. Co-Design Workshops

The co-design workshops were conducted to conceptualize the prototype and create a detailed mobile app interface. Based on the guidelines of Young and Well [12], workshops were conducted in the following sequence of six steps: (1) presenting a sketch of the co-designers’ idea of how the app should look in a storyboard format; (2) seeking feedback on each sketch from the co-designers; (3) identifying future users’ needs and picturing their needs through design cards and journey maps; (4) developing a group design for the content discussed in the previous steps; (5) confirming the most feasible interface design and developing it as an app prototype; and (6) discussing the app prototype in terms of revealing the developed content and usability, including effectiveness, efficiency, and engagement.

**Figure 1:**
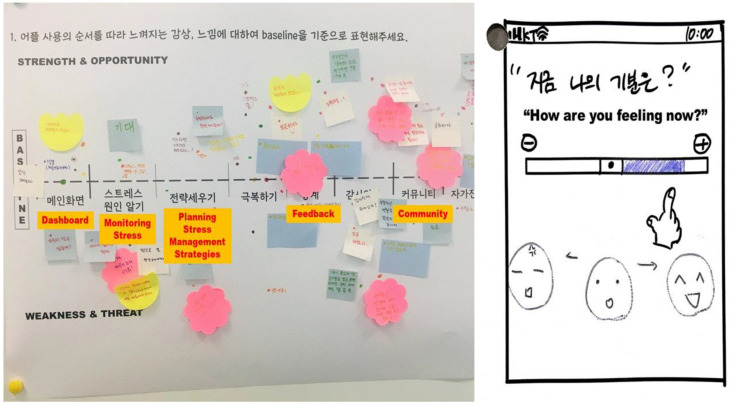
Design cards and journey maps in co-design workshops.

The PI, committee, and co-designers participated in the co-design workshops, which were held twice for two hours each. Eleven students were newly recruited as co-designers to conceptualize prototypes and create app pages that could be used more efficiently and would increase user immersion. These co-designers were expected to have similar characteristics as those who would actually use the developed app. The PI organized and monitored the workshops, and the committee acted as a facilitator to ensure that the workshop activities went well. During the workshops, the co-designers proposed various prototypes to reveal the four content components and provided feedback on the interface design, including layout, icons, and texts. For the app interface, the co-designers emphasized effectiveness, efficiency, and engagement. This was to make it easier for actual app users to understand and use the app, as well as continue using it. Interface designs reflecting co-designers’ needs were presented using storyboards, design cards, and journey maps (Figure 1).

### 2.2. Pilot Evaluation of the Developed App

Once the MEndorphins app for psychological distress management was developed as outlined in Section 2.1, the app’s usability, quality, and effects were evaluated, the details of which are summarized in this section.

#### 2.2.1. Study Design and Participants

This was a one-group, pretest-posttest design study. Participants were recruited from a university located in Seoul, South Korea between March and June 2020. The following inclusion criteria were used to identify eligible students: (1) aged between 19 and 25 years, 2) currently enrolled in a full-time bachelor’s degree program at a university, (3) user of a smartphone with Android (a mobile operating system by Google), and (4) able to use a smartphone without help from others. Based on power analysis calculations using G*power version 3.1, a sample size of at least 34 was needed to detect a medium effect size of 0.05, with a statistical power of 0.80 at an alpha level of 0.05 for analysis of pre-post mean differences in one group. A total of 50 university students were approached considering the dropout rate. Of these, 4 (8.0%) did not meet the inclusion criteria and 11 (22.0%) submitted incomplete post-test questionnaires. Finally, data from 35 participants (70.0%) was analyzed in this study.

#### 2.2.2. Measures

Usability: The ease of use of the developed app, MEndorphins, was measured using the Korean version of the System Usability Scale (SUS) [17]. The original SUS was developed to understand the problems that users face while using a system [18]. The SUS consists of 10 items on the usability of a system, which are rated on a five-point scale. The average SUS scores range between 0 and 100. Based on the guidelines offered by Bangor et al. [19], a score of 0–60 was considered not acceptable (0–50 = not acceptable, 50–60 = marginally not acceptable) and 60–100 was considered acceptable (60–70 = marginally acceptable, 70–100 = acceptable) for this study. Cronbach’s alpha for SUS in this study was 0.86.

Quality: The quality of MEndorphins was measured using the Korean version of the user version of the mobile application rating scale (uMARS) (2017). The original uMARS was developed to classify and assess the quality of mobile health apps [20]. The uMARS is a 26-item scale including 6 quality-dimension subscales: engagement (5 items), functionality (4 items), aesthetics (3 items), information (4 items), subjective quality (4 items), and other (6 items). The items are rated using a five-point Likert-type response scale. To calculate the mean quality score for each subscale, the scores of the individual items are averaged. The scores for four subscales (engagement, functionality, aesthetics, and information) are then averaged to obtain the total uMARS score. Cronbach’s alpha for uMARS in this study was 0.86.

Effects on psychological outcomes: Before and after their use of MEndorphins, students’ symptoms of depression, anxiety, and stress were measured using the validated Korean version of the Depression Anxiety Stress Scale (DASS) [21]. DASS is a 21-item self-report scale with depression, anxiety, and stress subscales [22]. Each subscale consists of seven items rated on a four-point scale. High scores indicate higher levels of depression, anxiety, and stress. Cronbach’s alpha for DASS in this study was 0.83 for depression, 0.85 for anxiety, and 0.77 for stress.

#### 2.2.3. Data Collection

The PI posted a participant recruitment notice online after obtaining permission from the university. Next, the PI approached willing students to assess their eligibility and interest in participating in the study. Students who provided written informed consent were invited to complete the pretest; they received a private code that allowed them to access and use MEndorphins. After the students had used the app for a week, frequency and usage time information was collected directly from the app. Students also completed an online survey to evaluate the usability and quality of MEndorphins and their effects on their psychological outcomes. This study was conducted between March and June 2020.

#### 2.2.4. Data Analysis

The data were analyzed using SAS version 9.3 (SAS Institute Inc., Cary, NC, USA). Descriptive statistics, including mean, median, standard deviation (SD), and percentages, were computed to summarize participant characteristics and survey scores. Additionally, paired t-tests were used to examine whether there was a difference in students’ depression, anxiety, and stress levels before and after using the app.

## 3. Results

### 3.1. Developed App

The app was developed based on BA techniques, which are composed of four main content components: monitoring, planning, feedback, and sharing. Through the prototype workshop, the interface design of the four MEndorphin content components was determined. The details are as follows.

Monitoring The stress monitoring page focused on making it easy to recognize the daily stress level. This section was designed as a self-report form that not only included a simple numeric scale, but also visualized colors or facial expressions. Under high-stress conditions, the color of the scroll bar, indicating the stress level, would turn red, and the face emoticon would show a depressed expression. Additionally, phrases such as “plan something” that tells nursing students whether they are in a stressful state was also presented.

Planning The planning page of the app focused on enabling nursing students to easily identify, plan, and check the effectiveness of their strategies. It included daily messages, such as “How is today progressing?” and “Start your planning by monitoring today’s stress levels” to motivate students to start the intervention. After monitoring their stress levels, nursing students would receive the message “Goal set–plan today’s stress management strategies” or “Goal achieved.” Students could start by choosing the category of coping strategies and then select up to three strategies to manage the day’s stressful situations. Icons and images of stress management strategies were presented so that students could easily identify their preferred strategies. They could also implement personally desired stress management strategies by entering specific strategies of their own.

Feedback The feedback page focused on encouraging nursing students to receive feedback on their stress coping strategies in various ways to inspire positive behavior. For this, a calendar feature was used to show three types of feedback: the duration of app usage, the number of days when strategic activities were completed, and whether the stress state was relieved. In addition, the frequency of implementing strategies was displayed on a pie chart, and the top three coping strategies, as rated by students, were presented with a gold-medal image.

Sharing The sharing page focused on helping nursing students effectively share their experiences of using the app with their peers so that they can obtain information on more diverse coping strategies and encourage each other to continue positive behaviors. An online board was created where students could post their reviews on app use and thoughts on effective stress coping strategies. A “like” button was also added based on the social networking website Facebook’s “like” feature.

**Figure 2:**
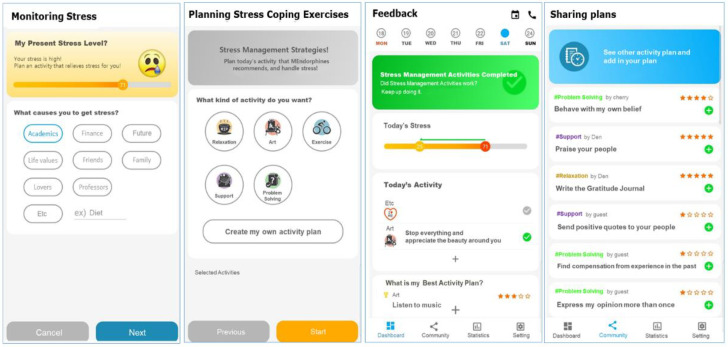
Developed app pages.

The content and interface design of the app were determined based on the results of the prototype workshops (Figure 2).

### 3.2. Sample Characteristics

A total of 35 students used the developed app, MEndorphins, for seven days. The mean age of the students was 21.7 years (SD = 1.7), and approximately two-thirds (66%) of them were female (Table 1). The median daily usage time of the app was 12.5 min (interquartile range [IQR] = 10, 20).

### 3.3. Usability

The average value of the total SUS score was 74.21 (SD = 13.99; see Table 2). The score was classified as “acceptable” based on the guidelines offered by Bangor et al. [19]. In the evaluation of each item, the items with a score of 4 or more were items 3 (“I thought the system was easy to use”), 7(“I believe that most people would learn to use this system very quickly”), and 9 (“I felt very confident using the system”).

### 3.4. Quality

The average value of the total uMARS score was 3.72 (SD = 0.36) out of 5 (Table 2). In the evaluation of each subscale, functionality was the dimension with the highest score (mean = 4.17). The average score for the perceived potential impact of the app was 3.98 out of 5.

### 3.5. Effects on Psychological Outcomes

There were no statistically significant differences in depression, anxiety, and stress between male and female students in the pretest. There were statistically significant changes in the scores of depression, anxiety, and stress symptoms after using MEndorphins (*p* ≤ 0.001 for depression and anxiety, *p* = 0.001 for stress; see Table 3). The students’ depressive symptoms decreased significantly (mean = 4.17, SD = 2.81), as did their anxiety (mean = 2.83, SD = 3.05) and stress symptoms (mean = 6.89, SD = 4.60) after using the app.

## 4. Discussion

This study aimed to develop an mHealth app for stress self-management interventions that college students can use easily and comfortably. To develop an app that people in their 20s can use on their own with interest, a co-design approach was used in which college students with characteristics similar to real users participated in the entire process from app development to evaluation.

The co-design approach allowed for the development of a user-friendly self-management app for college students, MEndorphins, which reflected real users’ perspectives and experiences. Because all the student committee members and co-designers are digital natives who can consume digital information and comfortably use mobile phones and apps [23], they were able to suggest enjoyable and customized apps for college students. The students demonstrated creative and innovative approaches while working together by using their knowledge and resources to achieve better outcomes in the development process. Customization is a significant strength of the co-design approach [24]. Specifically, the student committee and co-designers presented stressors that real users of the app could relate to and suggested easy and enjoyable stress-coping strategies. Therefore, the content of the self-management intervention reflected college students’ realities and was customized for them. In this way, the co-design approach provides opportunities for mental health service providers to understand what kind of stress real users experience and what strategies are needed.

The results of the evaluation of the use of MEndorphins suggest that an app with a BA approach can effectively manage psychological distress in college students. After using the app for seven days, perceived depression, anxiety, and stress symptoms significantly decreased. The results on the effects on psychological outcomes were consistent with the findings of previous studies on the efficacy of BA-based mHealth apps. In a review article, an mHealth app using BA as an intervention showed significant efficacy in reducing depression, anxiety, and stress [25]. In a study by Dahne et al. [26] in the US, a self-help mobile app (Moodivate) based on the BA approach was provided to adults who were being treated for depressive symptoms in a primary care setting. Moodivate applied BA approaches similar to the app in this study, and the participants were encouraged to use the app and complete the suggested activities for eight weeks. The analysis of changes in depressive symptoms over time indicated a significant decrease that was sustained throughout the trial period. In another study of an mHealth app using BA approaches, expected mastery or pleasure was found to correlate with an improvement in depression [27]. The efficacy of the BA approach reported in previous studies suggests that MEndorphins could be effective in managing psychological symptoms, such as depression, anxiety, and stress, among college students.

In terms of accessibility and availability, smartphone-based apps are a promising tool for the self-management of psychological distress symptoms. Participants in this study used MEndorphins 5.6 times a week on average. App users are not limited by time and place, and can access the app without revealing that they wish to receive help. According to Yoon’s study [28], 99.9% of those aged 20–49 years in South Korea own a smartphone. Considering the high penetration rate of smartphones, mHealth interventions can potentially be used to support college students and help reduce their psychological distress. Participants who used MEndorphins generally found it easy to use (74.2 out of 100 in SUS scores) and perceived the app to be of good quality (3.7 out of 5 in uMARS scores). Previous studies on mobile app development [29,30] have suggested that an app has an appropriate level of quality when the median value of the uMARS score is 2.9 or higher. It is difficult to directly compare the usability of the app developed in this study to those of other studies because of the different treatment approaches; however, MEndorphins satisfied or exceeded the usability and quality evaluation criteria suggested in previous studies.

There are some suggestions for future research. There are some suggestions for future research. In the co-design process for app development, it is important to integrate the views and expertise of all concerned stakeholders [31]. Therefore, future studies should consider including not only students but also clinicians in the co-design development process to include diverse views on stress management. For this study, participants who acted app development committee and co-designers were all recruited from a college in a metropolitan area. Participants in this study are limited in representing college students from other areas. Additionally, MEndorphins were developed based on the Android system and cannot be used with mobile phones using iOS or other operating systems. After confirming the effectiveness of the app, it will be necessary to develop it for other operating systems to expand its usability and help students manage their stress. This study could not show the long-term effects of app usage because the users used MEndorphins only for seven days. To examine the long-term effects of app use, it is necessary to expand the duration of app use and evaluate changes in psychological distress in future studies.

Despite these limitations, the study findings show that BA-based MEndorphins could be a therapeutic self-management tool to relieve psychological distress in college students. The app developed in this study is different from the existing ones in that it provides brief personalized interventions designed to support young people. MEndorphins users can monitor their mood, plan stress self-management strategies, and gain motivation by effectively sharing their experiences.

## 5. Conclusions

The mobile app developed in this study, MEndorphins, is meaningful because it involved real users, college students, in both the app development and evaluation process. Moreover, the BA interventions proposed as a strategy for stress management in MEndorphins are expected to help college students to strengthen their independent coping skills easily and comfortably. MEndorphins can be also incorporated into existing face-to-face mental health support services for young people. In future research, it is needed to investigate the opinions on the feasibility and acceptability of app targeting college students from various regions, and to explore whether the developed app have long-term effects as well as short-term effects on their psychological distress.

## Figures and Tables

**Table 1:** Characteristics of participants (N = 35).

Characteristic		n (%)	Mean (SD)	Median	IQR [Q1–Q3]
Gender					
	Male	12 (34.29)			
	Female	23 (65.71)			
Age			21.66 (1.70)		
Usage time (min/day)					
	<10	17 (48.57)	28.53 (48.81)	12.50	10.0 [10–20]
	10–30	14 (40.0)			
	>30	4 (11.43)			
Usage frequency (per week)					
	1–5	17 (48.6)	5.6 (2.17)	4	3 [4–7]
	>5	18 (51.4)			

SD = Standard deviation, IQR = Interquartile range.

**Table 2:** Perceived usability and quality of the developed application.

Category	Mean (SD)
Usability	74.21 (13.99)
Quality	
App quality ^a^	3.72 (0.36)
Engagement	3.15 (0.44)
Functionality	4.17 (0.48)
Aesthetics	3.67 (0.60)
Information	3.88 (0.54)
App subjective quality	2.99 (0.61)
Perceived impact of application use	3.98 (0.46)

SD = standard deviation, ^a^ = (engagement + functionality + aesthetics + information)/4.

**Table 3:** Effects of application use on psychological outcomes.

Variables	Mean (SD)		t	*p*	95% CI
	Pre	Post			
Depression	5.89 (4.16)	4.17 (2.81)	2.75	<0.001	0.45 to 2.99
Anxiety	4.66 (3.76)	2.83 (3.05)	2.94	<0.001	0.56 to 2.98
Stress	9.94 (5.70)	6.89 (4.60)	4.27	0.001	1.60 to 4.51

SD = Standard deviation, CI = Confidence interval.

## Data Availability

The data presented in this study are available on request from the corresponding author. The data are not publicly available due to privacy or ethical restriction.

## References

[1] (2016). Seoul Mental Health Statistics. Mental Health Statistics. https://seoulmentalhealth.kr/.

[2] Cuijpers P., Cristea I.A., Ebert D.D., Koot H.M., Auerbach R.P., Bruffaerts R., Kessler R.C. (2016). Psychological Treatment of Depression in College Students: A Metaanalysis. Depress. Anxiety.

[3] Ribeiro Í.J.S., Pereira R., Freire I.V., de Oliveira B.G., Casotti C.A., Boery E.N. (2018). Stress and Quality of Life Among University Students: A Systematic Literature Review. Health Prof. Educ..

[4] Sharp J., Theiler S. (2018). A Review of Psychological Distress Among University Students: Pervasiveness, Implications and Potential Points of Intervention. Int. J. Adv. Couns..

[5] Lee R.A., Jung M.E. (2018). Evaluation of an MHealth App (Destressify) on University Students’ Mental Health: Pilot Trial. JMIR Ment. Health.

[6] Situmorang D.D.B. (2020). Online/Cyber Counseling Services in the COVID-19 Outbreak: Are They Really New?. J. Pastoral Care Couns..

[7] Rodriguez-Paras C., Tippey K., Brown E., Sasangohar F., Creech S., Kum H.C., Lawley M., Benzer J.K., Benzer J.K. (2017). Posttraumatic Stress Disorder and Mobile Health: App Investigation and Scoping Literature Review. JMIR mHealth uHealth.

[8] Wang K., Varma D.S., Prosperi M. (2018). A Systematic Review of the Effectiveness of Mobile Apps for Monitoring and Management of Mental Health Symptoms or Disorders. J. Psychiatr. Res..

[9] Dimidjian S., Barrera Jr. M., Martell C., Muñoz R.F., Lewinsohn P.M. (2011). The Origins and Current Status of Behavioral Activation Treatments for Depression. Annu. Rev. Clin. Psychol..

[10] Hopko D.R., Lejuez C.W., Ruggiero K.J., Eifert G.H. (2003). Contemporary Behavioral Activation Treatments for Depression: Procedures, Principles, and Progress. Clin. Psychol. Rev..

[11] Ekers D., Webster L., Van Straten A., Cuijpers P., Richards D., Gilbody S. (2014). Behavioural Activation for Depression; an Update of Meta-Analysis of Effectiveness and Sub Group Analysis. PLoS ONE.

[12] Hagen P., Collin P., Metcalf A., Nicholas M., Rahilly K., Swainston N. (2012). Participatory Design of Evidence-Based Online Youth Mental Health Promotion, Intervention and Treatment. https://www.westernsydney.edu.au/__data/assets/pdf_file/0005/476330/Young_and_Well_CRC_IM_PD_Guide.pdf.

[13] Chon K., Kim K.H., Yi J.S. (2000). Development of the Revised Life Stress Scale for College Students. Korean J. Health Psychol..

[14] Lee E.H., Kim S.J. (2012). Validity and Application of the Life Stress Scale for University Students. J. Educ. Res..

[15] Davis M., Eshelman E.R., McKay M. (2008). The Relaxation and Stress Reduction Workbook.

[16] Polit D.F., Beck C.T. (2006). The Content Validity Index: Are You Sure You Know What’s Being Reported? Critique and Recommendations. Res. Nurs. Health.

[17] Jeong Y.W., Chang H.J., Kim J.A. (2020). Development and Feasibility of a Safety Plan Mobile Application for Adolescent Suicide Attempt Survivors. Comput. Inform. Nurs..

[18] Brooke J., Jordan P.W., Thomas B., McClelland I.L., Weerdmeester B. (1996). SUS: A ‘Quick and Dirty’ Usability Test. Usability Evaluation in Industry.

[19] Bangor A., Kortum P., Miller J. (2009). Determining What Individual SUS Scores Mean: Adding an Adjective Rating Scale. J. Usability Stud..

[20] Stoyanov S.R., Hides L., Kavanagh D.J., Wilson H. (2016). Development and Validation of the User Version of the Mobile Application Rating Scale (uMARS). JMIR mHealth uHealth.

[21] Lee E.H., Moon S.H., Cho M.S., Park E.S., Kim S.Y., Han J.S., Cheio J.H. (2019). The 21-Item and 12-Item Versions of the Depression Anxiety Stress Scales: Psychometric Evaluation in a Korean Population. Asian Nurs. Res..

[22] Henry J.D., Crawford J.R. (2005). The Short-Form Version of the Depression Anxiety Stress Scales (DASS-21): Construct Validity and Normative Data in a Large Non-Clinical Sample. Br. J. Clin. Psychol..

[23] Evans C., Robertson W. (2020). The Four Phases of the Digital Natives Debate. Hum. Behav. Emerg. Technol..

[24] Grant C., Widnall E., Cross L., Simonoff E., Downs J. (2020). Informing the Development of an E-Platform for Monitoring Wellbeing in Schools: Involving Young People in a Co-Design Process. Res. Involv. Engagem..

[25] Khademian F., Aslani A., Bastani P. (2020). The Effects of Mobile Apps on Stress, Anxiety, and Depression: Overview of Systematic Reviews. Int. J. Technol. Assess. Health Care.

[26] Dahne J., Lejuez C.W., Diaz V.A., Player M.S., Kustanowitz J., Felton J.W., Carpenter M.J. (2019). Pilot Randomized Trial of a Self-Help Behavioral Activation Mobile App for Utilization in Primary Care. Behav. Ther..

[27] Furukawa T.A., Imai H., Horikoshi M., Shimodera S., Hiroe T., Funayama T., Akechi T., FLATT Investigators (2018). Behavioral Activation: Is It the Expectation or Achievement, of Mastery or Pleasure That Contributes to Improvement in Depression?. J. Affect. Disord..

[28] Yoon J.S. (2021). Smartphone Ownership South Korea 2020, by Age Group. https://www.statista.com/statistics/897195/south-korea-smartphone-ownership-by-age-group/.

[29] Mani M., Kavanagh D.J., Hides L., Stoyanov S.R. (2015). Review and Evaluation of Mindfulness-Based IPhone Apps. JMIR mHealth uHealth.

[30] McKay F.H., Wright A., Shill J., Stephens H., Uccellini M. (2019). Using Health and Well-Being Apps for Behavior Change: A Systematic Search and Rating of Apps. JMIR mHealth uHealth.

[31] Ward M.E., De Brún A., Beirne D., Conway C., Cunningham U., English A., Fitzsimons J., Furlong E., Kane Y., Kelly A. (2018). Using Co-Design to Develop a Collective Leadership Intervention for Healthcare Teams to Improve Safety Culture. Int. J. Environ. Res. Public Health.

